# After a patient dies by suicide: an illustrative case for trainee psychiatrists and trainers

**DOI:** 10.1192/bjb.2021.106

**Published:** 2022-10

**Authors:** Alice Oates, Rachel Gibbons

**Affiliations:** 1 Southern Health NHS Foundation Trust, Basingstoke, UK; 2 Royal College of Psychiatrists, London, UK

**Keywords:** Suicide, education and training, supervision, trauma, self-harm

## Abstract

One of the most challenging experiences psychiatrists will face in their careers may well be the death of a patient by suicide. This is likely to happen at least once during a psychiatrist's career, and often more. It can be an intensely complex and painful event with a wide range of emotional responses. Reflecting on the death and accessing good support helps clinicians process the emotional impact. It can also increase their resilience in the longer term by giving them a greater understanding of both their own and their patients’ limitations, and in this way strengthen their capacity for compassion as clinicians. Using an illustrative case study, this article provides an insight into the experience of losing a patient to suicide and signposts to sources of support.

## Clinical scenario

Dr Day is a higher trainee on call covering a busy emergency department. He has been asked by one of the psychiatric liaison nurses working in the department to review Miss B, a 23-year-old female who has taken an overdose of paracetamol and codeine following an argument with her partner. Miss B had been drinking alcohol but is now sober and agreeing to speak to the mental health team.

Dr Day reviews Miss B, who reports that this is the first time she has taken an overdose. She has been having arguments with her partner, is concerned that the relationship is about to end and feels that things have ‘just become too much’. Miss B reports that the overdose was impulsive and influenced by her intoxication at the time. Although she did not alert anyone to the overdose, her partner found her unconscious and called an ambulance. Dr Day also takes a collateral history from her mother, but Miss B declines a request to speak to her partner. During the assessment, there is no indication that Miss B has a current mood or psychotic disorder.

Miss B reports that she now regrets having taken the tablets and reports no thoughts or plans to harm herself again. Blood test results indicate that she does not require any medical treatment for the overdose. She is keen to leave the department and assures Dr Day that she will be staying with her mother for a couple of days. Her mother is supportive of this plan. Dr Day considers follow-up with the patient – however, it is not felt that any ongoing mental health input is required. Before Miss B leaves, Dr Day collaboratively develops a safety plan with her, including recognition of things she is motivated to achieve in the future, distraction techniques she identifies as helpful, agreement with Miss B and her mother that medication will not be routinely kept in the house, provision of the healthcare trust's crisis line number, and information about relationship counselling and local talking therapy services. Dr Day also writes to Miss B's general practitioner with the details of the emergency department attendance.

Two weeks later, Dr Day is called to see his clinical supervisor and is informed that the patient has been found deceased, at home. It is likely that she died by suicide.

Dr Day was shocked by the sad news. He had not been expecting the death. Over the next few days, despite not having thought about the patient since she was discharged, Dr Day began to replay the events in his mind, constructing a narrative where he has missed something important. Dr Day felt incredibly anxious that he had been the last doctor to see the patient and that he was in some way to blame for her death. Quite quickly, these feelings of guilt escalated to the point where Dr Day was frequently tearful, became fearful of patient contact and dreaded being on call. He felt extremely isolated and, following much internal debate, took a week off work.

## Epidemiology of patient suicide

Although the exact figure is unknown, it is estimated that 51–82% of psychiatrists experience at least one patient suicide in the course of their career,^1^ and for some the number is higher.^2^ It is likely that psychiatric trainees will either have personal experience or know colleagues who have lost a patient to suicide. Even with good clinical care, some patients die in this way. It is important to remember that around 75% of those who die by suicide have not been under the care of mental health services in the year before their death.^3^ One theory is that suicide can be a response to a loss and represents a difficulty in mourning.^4,5^ Suicide prevention is therefore a public health issue and there is good evidence that public health interventions are the best way of preventing suicide.^6^ In light of the uncertainty and difficulty in knowing which patients are at particular risk of suicide it can be tempting to reach for an algorithm or flowchart for prediction. This has been shown to be inadequate and there is clear evidence now that risk assessment tools do not accurately predict the risk of suicide.^3,6–9^ There is emerging evidence that a good psychosocial assessment, the appropriate management of any identified risks and safety planning with the patient are effective in reducing suicidal behaviour,^6,10^ although none of these methods are 100% effective at preventing suicide.

## The effect of patient suicide on clinicians

It is common for psychiatrists to feel shocked and upset, with other emotions also emerging, including guilt, anger, anxiety and shame. Fear of judgement and blame by other colleagues is also frequent.^11^ Many psychiatrists report that they have felt a degree of responsibility and have believed that in some way their decision-making contributed to the death.^2^ The feeling of shame can be damaging, resulting in emotional withdrawal from possible sources of support. Research into the impact of suicide on mental health staff suggests that the effect can be comparable to that of other traumatic life events such as the death of a parent,^12–16^ and that active monitoring for symptoms of post-traumatic stress disorder may be appropriate.^17^

The intensity of emotion felt following the death of a patient can be great and will vary from clinician to clinician. It is currently unclear why some deaths have a greater impact than others and affect some of the clinicians involved in the care more than other clinicians. Some speculated factors include: the clinician's relationship with the patient, whether they were the last clinician to have contact and their personal vulnerability at the time of the death. What is clear is that the support received following a patient suicide is critical to the subsequent response.^14,18–22^ A recognition of this is leading to the development of various postvention protocols for mental health staff.^18–22^

## Advice for clinicians

No individual can be to blame for anyone's death by suicide. Blame implies a single causal factor and that the responsibility for the death lies with one person only. This is clearly not the case and denies the complex reality of suicide. Although doctors may have some responsibility for an aspect of the care provided, or not provided, to assume too much responsibility is not reasonable.

It is important to remember that all staff groups, including those working in non-clinical areas, may be affected by a patient suicide and different individuals are likely to require different types of support at different times.^23^ Trainees may not be in a position to offer this help directly, but an awareness of the needs of other team members is important. After the death there is likely to be a multidisciplinary team debrief with a pastoral focus to discuss what was known about the patient and the care that was provided and to offer some space for emotional responses. These meetings are often facilitated by a psychologist. Generally, all those who have been involved in the care are invited. This invite should include those not currently attached to the team. These meetings can be challenging but are generally found to be helpful.^24^ It is important to recognise that some clinicians may feel too emotionally distressed to be able to attend, or contribute, at this early stage.^24^

The Patient Safety Group of the Royal College of Psychiatrists offers practical guidance in the College Report *Self-harm and Suicide in Adults*.^6^ This report highlights the need to have established support structures in place for all staff, including those in training, accessible when the need arises. It also recommends regular reflective space (such as Balint groups or reflective practice groups) as part of everyday work.

The gap in consistent support for clinicians after the death of a patient by suicide has recently been addressed by the Oxford Centre for Suicide Research in the booklet *If a Patient Dies by Suicide: A Resource for Psychiatrists*.^11^ In this guide, psychiatrists are encouraged to prioritise self-care and connecting with others and to try to avoid the often inaccurate narratives developing around the death. The booklet also normalises the experiences of others who have gone through this process, with the aim of supporting both trainees and qualified psychiatrists when a patient suicide occurs.

## Supporting the family

Dr Day and his supervisor met with a senior member of the psychiatric liaison service who knew about the death and had met with Miss B's mother to offer condolences. Dr Day was particularly concerned for the well-being of Miss B's mother and felt he wanted to offer her support. It was decided that that it was not in the best interests of Dr Day or Miss B's mother for them to meet at this point. Miss B's mother's grief was very raw and Dr Day was advised that he might have difficulty coping due to his current vulnerability. He was reassured that bereavement support had been provided and that there would shortly be a formal meeting with the family and those involved in the care of Miss B, and that they would be given the opportunity to contribute to the serious incident investigation should they wish.

### Support available for families and peers

Deaths by suicide often lead to complex bereavements and families will understandably vary in their ability to be involved in interviews and investigations.^25^ Offering supportive information, such as Public Health England's *Help is at Hand* booklet,^26^ can provide some comfort and start the process of engagement. Organisations will have formal support structures, and the responsibility for communicating and supporting the family is a systemic one. This should not be left to individual clinicians involved in the care of the patient, who are likely to be emotionally affected themselves. Some mental health organisations have started to adopt a family liaison service model with which to support bereaved families following a death.^27^

There is growing recognition that the death of a patient may have a significant impact on other patients who have known the deceased in a variety of contexts. Although research is limited, it is generally agreed that the vulnerability of other patients may be increased following a peer suicide and that they may need additional support at this time.^28^

## Investigations and inquests

### Internal investigation

An internal investigation follows most deaths of patients under the care of mental health services. This occurs before the coroner's inquest, meaning that at the time of the investigation the cause of death (and whether or not it is death by suicide) has not yet been confirmed. The aim of the investigation is to examine the care pathway provided to the patient who has died, so that any learning from this can contribute to future service development. The aim is not to determine what led to the death, as that can only be hypothetical at this stage.

There is a compassionate and legal obligation, called the Duty of Candour,^29^ to involve families and be open and honest in this investigation. It is important that families are involved as they have information pertinent to the investigation and may have concerns about care that can be addressed during this process. It has been shown that suicide prevention and care for patients improve in mental health organisations that routinely involve families in clinical care and organisational processes.^3^

Routinely, clinicians who have been involved in the patient's care are interviewed as part of this investigation. Clinicians may feel understandably anxious about this. However, anecdotaly many find the process helpful as it provides them with space and support to discuss care decisions. During the process, clinicians may also find out more about the wider circumstances of the death. It is important that trainees feel encouraged to invite their clinical or educational supervisor to attend for support.

After the internal investigation, a serious incident report will be provided to the coroner to assist the inquest. The principles that underpin an investigation, as well as an explanation of the investigation process, can be found in NHS England's Serious Incident Framework (SIF) policy.^30^

### Coroner's inquest

The role of the coroner is not to apportion blame or to guess at the events that contributed to the death, but to determine who has died, how they died, and where and when they died. From the wide range of evidence provided it may become clear that the care provided by mental health services formed only a small part of the individual's life. Many people find that the experience of attending the inquest gives some sense of resolution. Nevertheless, even the most experienced doctors can become anxious at the thought of being summoned.

Coroners’ courts are ‘open hearings,’ meaning that, as well as the professionals involved in delivering care, members of the public (including relatives and friends) can attend. There is likely to be a range of emotional response from those hearing and giving evidence.

Many deaths by suicide occur despite high-quality practice and the best efforts of those providing care. However, there are times when problems with service delivery or clinical practice are highlighted by the internal investigation or the coroner's inquest. Any problems identified should be systemic in focus and not personally directed towards any individual. If the coroner becomes concerned about particular care practices and believes that urgent action needs to be taken to reduce the risk of further loss of life, they can issue a Prevention of Future Deaths (PFD) notice. This requires the respondent to reply within 56 days with the proposed changes in care that will address the issue.^31^ An example of such a PFD and the responses to it are available online.^32^

## Training and practical issues

Dr Day took the experience to his Balint group and was able to discuss with other trainees both the case itself and his response to it. He found this process extremely useful in understanding some of the things that he had been feeling, recognising that other trainees, who he considered competent and professional, had also had similar experiences and reactions.

Dr Day was able to write a reflection in his portfolio where he described and reviewed the incident and the journey he had been through in terms of learning and recovery. Dr Day highlighted this reflection in his Annual Review of Competence Progression (ARCP) documentation and was praised by the ARCP panel for offering such a considered and insightful response.

## Trainees’ responsibilities

It is likely that trainees will have deep personal reflections, over the many years following the death, if involved in a case similar to the one outlined here. A patient suicide would be regarded as a ‘serious incident’ and as such will need to be recorded appropriately on ARCP documentation submitted to the deanery. Patient deaths by suicide are a part of the experience of being a psychiatrist and recording them does not affect progress through training. Not disclosing incidents may lead to questions regarding probity – if trainees are ever unsure about what information to document they can confirm this with their deanery or clinical and educational supervisors.

## Guidance for supervisors

As a trainer or supervisor, it can be difficult to know how much support to offer a trainee, particularly if the trainer or supervisor has had difficult past experiences with patient suicide and limited support themselves. For the coroner's inquest, it is important that trainers and managers take care of logistical issues such as guaranteeing that any trainees involved have the day off work, are not on call that evening and are not coming off a night shift. It may be that a supervisor hears about an incident before a trainee does. In this situation, it is important that they inform their trainee face to face and at a time where a supportive discussion can take place. This prevents trainees having the shocking, but not atypical, experience of receiving an unexpected message notifying them about the death of a patient or asking them to attend an investigation. It is useful to point them in the direction of the booklet mentioned earlier (*If a Patient Dies by Suicide*)^11^ and other resources available on the Royal College of Psychiatrists’ website, where they can also watch videos of psychiatrists talking about their own experiences following a patient death of this nature.^33^ It is very valuable for supervisors to make themselves available to support the trainee on the day of the inquest and to ‘check in’ with them periodically afterwards, monitoring them for signs that they are not coping, such as increased sickness, reduced performance and greater apparent emotional vulnerability.

## Conclusions

Discovering that a patient has died by suicide, and the process that follows, can be emotionally stressful and traumatic for clinicians involved in the patient's care. It is hoped that this illustrative case helps clinicians prepare themselves for how they might respond to the suicide of a patient whose care they have been involved in, and how they might best support others in that situation. It should also help clinicians going through this unfortunate process at the moment. Many clinicians have had this experience and it is important to challenge unhelpful narratives and to ask for help and support. St John-Smith et al offer guidance for psychiatrists on preparing for a coroner's inquest following a patient's death^34^ and NHS Resolution has published guidance on supporting staff to prepare for inquests.^35^ Other helpful resources may be found on the Royal College of Psychiatrists’ website.^33^

## Data Availability

Data availability is not applicable to this article as no new data were created or analysed in this study.

## About the authors

**Alice Oates** is a Specialty Trainee Year 6 (ST6) in general adult psychiatry currently working in liaison psychiatry within Southern Health NHS Foundation Trust at Basingstoke and North Hampshire Hospital, Basingstoke, UK. **Rachel Gibbons** is a consultant psychiatrist. She works independently. She also practises as a psychoanalyst and group analyst. She is Chair of the Royal College of Psychiatrists’ Patient Safety Group and the College's Working Group on the Effect of Suicide and Homicide on Psychiatrists.

## Supplementary material

For supplementary material accompanying this paper visit http://doi.org/10.1192/bjb.2021.106. click here to view supplementary material

## References

[1] Séguin M, Bordeleau V, Drouin MS, Castelli-Dransart DA, Giasson F. Professionals’ reactions following a patient's suicide: review and future investigation. Arch Suicide Res 2014; 18: 340–62. 24846577 10.1080/13811118.2013.833151

[2] Gibbons R, Brand F, Carbonnier A, Croft A, Lascelles K, Wolfart G, Effects of patient suicide on psychiatrists: survey of experiences and support required. BJPsych Bull 2019; 43: 236–41.

[3] National Confidential Inquiry into Suicide and Safety in Mental Health. Safer Services: A Toolkit for Specialist Mental Health Services and Primary Care. University of Manchester, 2021 (https://documents.manchester.ac.uk/display.aspx?DocID=40697).

[4] Campbell D, Hale R. Working in the Dark: Understanding the Pre-Suicide State of Mind. Routledge, 2017.

[5] Gibbons R, Adshead G. Psychodynamic aspects of suicide and homicide. In Seminars in the Psychotherapies (2nd edn) (eds R Gibbons, J O'Reilly): 247–58. Cambridge University Press, 2021.

[6] Royal College Psychiatrists. Self-Harm and Suicide in Adults: Final Report of the Patient Safety Group (College Report CR229). RCPsych, 2020.

[7] Rahman M, Kapur N. Quality of risk assessment prior to suicide and homicide. Psychiatr Bull 2014; 38: 46–7. 10.1192/pb.38.1.46b PMC4067846 25237494

[8] Windfuhr K, Kapur N. Suicide and mental illness: a clinical review of 15 years findings from the UK national confidential inquiry into suicide. Br Med Bull 2011; 100: 101–21. 21948337 10.1093/bmb/ldr042

[9] National Institute for Health and Care Excellence. Self-Harm in Over 8s: Long-Term Management (Clinical Guideline CG133). NICE, 2019 (https://www.nice.org.uk/guidance/cg133 [cited 16 Jun 2021]). 31891461

[10] Nuij C, Van Ballegooijen W, De Beurs D, Juniar D, Erlangsen A, Portzky G, Safety planning-type interventions for suicide prevention: meta-analysis. Br J Psychiatry 2021; 219: 419–26. 35048835 10.1192/bjp.2021.50

[11] University of Oxford Centre for Suicide Research. If a Patient Dies by Suicide: A Resource for Psychiatrists. Centre for Suicide Research, 2020 (https://www.rcpsych.ac.uk/members/supporting-you/if-a-patient-dies-by-suicide [cited 7 Jan 2020]).

[12] Chemtob C, Hamada RS, Bauer GB, Kinney B, Torigoe R Patients’ suicide: frequency and impact on psychiatrists. Am J Psychiatry 1998; 145: 224–8. 10.1176/ajp.145.2.224 3341466

[13] Chemtob C, Hamada RS, Bauer GB, Kinney B, Torigoe R Patient suicide: frequency and impact on psychologists. Prof Psychol Res Pr 1988; 19: 416–20.

[14] Castelli Dransart DA, Heeb JL, Gulfi A, Gutjahr EM. Stress reactions after a patient suicide and their relations to the profile of mental health professionals. BMC Psychiatry 2015: 265. 26511910 10.1186/s12888-015-0655-y PMC4624606

[15] Ruskin R, Sakinofsky I, Bagby RM, Dickens S, Sousa G. Impact of patient suicide on psychiatrists and psychiatric trainees. Acad Psychiatry 2004; 28: 104–10. 15298861 10.1176/appi.ap.28.2.104

[16] Valente S. Psychotherapist reactions to the suicide of a patient. Am J Orthopsychiatry 1994; 64: 614–21. 7847577 10.1037/h0079574

[17] Sandford DM, Kirtley OJ, Thwaites R, O'Connor R. The impact on mental health practitioners of the death of a patient by suicide: a systematic review. Clin Psychol Psychother 2021; 28: 261–94. 10.1002/cpp.2515 32914489

[18] Whisenhunt JL, DuFresne RM, Stargell NA, Rovnak A, Zoldan CA, Kress VE. Supporting counselors after a client suicide: creative supervision techniques. J Creativ Men Health 2017; 12: 451–67.

[19] Leaune E, Cuvillier B, Vieux M, Pacaut-Troncin M, Chalancon B, Perez A-F, The SUPPORT-S protocol study: a postvention program for professionals after patient or user suicide. Front Psychol 2020; 11: 805. 32431643 10.3389/fpsyg.2020.00805 PMC7217323

[20] Tedeschi RG, Calhoun LG. The Posttraumatic Growth Inventory: measuring the positive legacy of trauma. J Trauma Stress 1996; 9: 455–71. 8827649 10.1007/BF02103658

[21] Gutin NJ. Losing a patient to suicide: what we know. Curr Psychiatry 2019; 18: 15–32.

[22] Leaune E, Ravella N, Vieux M, Poulet E, Chauliac N, Terra JL. Encountering patient suicide during psychiatric training: an integrative, systematic review. Harv Rev Psychiatry 2019; 27: 141–9. 31082992 10.1097/HRP.0000000000000208

[23] Takahashi C, Chida F, Nakamura H, Akasaka H, Yagi J, Koeda A, The impact of inpatient suicide on psychiatric nurses and their need for support. BMC Psychiatry 2011; 11: 38. 21385448 10.1186/1471-244X-11-38 PMC3063822

[24] Alexander D, Klein S, Gray NM, Dewar IG, Eagles JM. Suicide by patients: questionnaire study of its effect on consultant psychiatrists. BMJ 2020; 320: 1571–4. 10.1136/bmj.320.7249.1571 PMC27400 10845964

[25] Spillane A, Matvienko-Sikar K, Larkin C, Corcoran P, Arensman E. What are the physical and psychological health effects of suicide bereavement on family members? An observational and interview mixed-methods study in Ireland. BMJ Open 2018; 8(1): e019472. 10.1136/bmjopen-2017-019472 PMC5781012 29331974

[26] Public Health England, Support after Suicide Partnership. Help is at Hand: Support after Someone May Have Died by Suicide. PHE, 2015 (https://supportaftersuicide.org.uk/resource/help-is-at-hand/ [cited 7 Jan 2021]).

[27] Oates A. Learning from suicide related claims. *NHS Resolution*, 2018 (https://resolution.nhs.uk/wp-content/uploads/2018/09/NHS-Resolution_learing_from_suicide_claims_148pp_ONLINE1.pdf [cited 30 Oct 2021]).

[28] Seeman M. The impact of suicide on co-patients. Psychiatr Q 2015; 86: 449–57. 25618004 10.1007/s11126-015-9346-6

[29] Nursing and Midwifery Council, General Medical Council. Openness and Honesty when Things go Wrong: The Professional Duty of Candour. GMC/NMC, 2019.

[30] NHS England Patient Safety Domain. *Serious Incident Framework*: Supporting Learning to Prevent Recurrence. NHS England, 2015 (https://www.england.nhs.uk/wp-content/uploads/2020/08/serious-incidnt-framwrk.pdf [cited 21 Jun 2021]).

[31] Courts and Tribunals Judiciary. Coroners (Investigations) Regulations 2013. Judiciary, 2021 (https://www.legislation.gov.uk/uksi/2013/1629/pdfs/uksi_20131629_en.pdf [cited 16 Jun 2021]).

[32] HM Assistant Coroner David Reid on behalf of the Courts and Tribunals Judiciary. Regulation 28: Report to Prevent Future Deaths. 2019 (https://www.judiciary.uk/publications/sasha-forster/ [cited 21 Jun 2021]).

[33] Royal College of Psychiatrists. If a patient dies by suicide. RCPsych, 2021 (https://www.rcpsych.ac.uk/members/supporting-you/if-a-patient-dies-by-suicide [cited 21 Jun 2021]).

[34] St John-Smith P, Michael A, Davies T. Coping with a coroner's inquest: a psychiatrist's guide. Adv Psychiatr Treat 2009; 15: 7–16.

[35] NHS Resolution. Inquests: A Guide for Health Providers. Supporting Staff to Prepare for an Inquest. NHS Resolution, 2020 (https://resolution.nhs.uk/wp-content/uploads/2020/03/Inquests-films-and-guide.pdf [cited 21 Jun 2021]).

